# A case of pelvic actinomycosis affecting genital, urinary and digestive tracts: a rare misleading diagnosis

**DOI:** 10.2144/fsoa-2021-0032

**Published:** 2021-06-22

**Authors:** Bernard Najib, Wissam Arab, Joseph Khazen, Yara Abdelkhalek, Wael Abdallah, Abir Khaddage, David Atallah

**Affiliations:** 1 Department of Obstetrics & Gynecology, Saint Joseph University, Beirut, Lebanon; 2 Department of Pathology, Saint Joseph University, Beirut, Lebanon

**Keywords:** actinomycosis, antibiotic therapy, culture, cystectomy, intrauterine device, malignancy, pelvic, rectosigmoid, sinus tract, surgery

## Abstract

Pelvic actinomycosis is an uncommon chronic invasive disease caused by a bacteria of the *Actinomyces spp.* Its diagnosis constitutes a clinical challenge and is usually reached in the postoperative period after resecting a pelvic mass that usually mimics advanced ovarian cancer. Although pelvic actinomyocosis involving the digestive and genital tract has been commonly described, very few reports have described cases involving both ovaries and requiring partial cystectomy for bladder involvement. Herein, we illustrate a case of pelvic actinomycosis with extensive involvement of multiple pelvic organs, misleading the surgeon into undergoing a complete clearance of the wrongfully thought adnexal malignancy.

## Lay abstract

Actinomycosis is an infection that affects the genital tract and is usually related to the presence of a foreign body on which the bacteria develops and then spreads to organs of the genital, urinary and digestive system. The infection can spread through organs by destroying and infiltrating the anatomical limits between them. Physicians are prone to performing a resection of these infiltrative lesions for fear of cancer. However, the disease can be simply cured by prescribing antibiotics for a prolonged period of time. New techniques can be used to identify the germ and prevent physicians from performing unnecessary resections.

Actinomycosis is a chronic infectious disease, caused by a Gram-positive anaerobic bacteria of the genus *Actinomyces*, with *Actinomyces israelii* being the most prevalent species [1]. The bacterium is endogenous: it exists within the oral flora and is also a common inhabitant of the female genital tract. Therefore, the infection is considered opportunistic and requires a breach in the mucosal lining, since this constitutes the first line of defense against the germ. After gaining access to deeper tissues with impaired blood supply, the germs proliferate in the form of branching filamentous rods, producing suppurative abscesses and/or granulomas. The infection then spreads by direct extension using sinus tracts and can involve other organs in proximity with the primary entry site [1].

The three main clinical types of actinomycosis with order of frequency are the cervicofacial, abdominopelvic and thoracic infections [2]. At the end of the 20th century, it was thought that *Actinomyces* can rarely involve the genitourinary system. However, recent reports and literature reviews are showing a higher prevalence of pelvic infections.

We hereby illustrate a case of pelvic actinomycosis in a 49-year-old female, that was attributed to a forgotten intrauterine device (IUD), with delayed diagnosis despite laparoscopic assessment. The tubo-ovarian infection in this case was bilateral and direct extension into the bladder dome and the rectal wall had occurred, requiring extensive surgical resection.

## Case report

**Figure 1.**
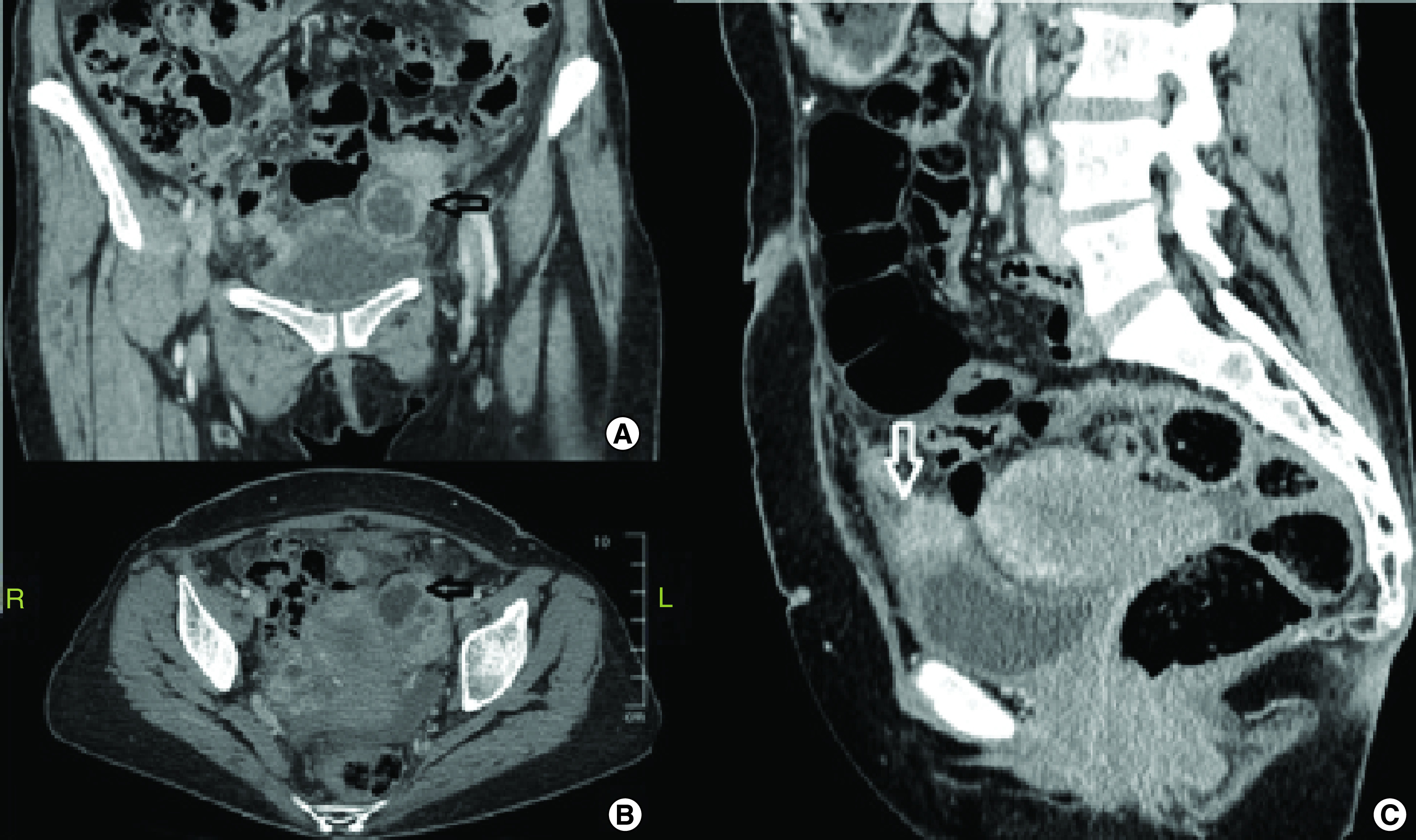
Pelvic computed tomography scan images during first emergency admission. **(A & B)** Contrast enhanced computed tomography showing the conglomerated mass around the left adnexa filling the pelvis on coronal and axial views. **(C)** Enhanced 2 cm implant lying on the bladder dome and infiltrating its wall with a bulging effect.

A 49-year old perimenopausal Caucasian woman, *gravida* 3 *para* 3, was referred to our institution for a suspicious adnexal lesion. She complained of an intermittent pelvic pain over the preceding 3 months accompanied by weight loss. No fever was reported, neither bloody nor purulent vaginal discharge. Physical examination showed a mild tenderness at the lower quadrants without a palpable adnexal mass. Gynecological exam did not find signs of inflammation in the vaginal or cervical mucosa. No cervical motion tenderness was found. Patient’s medical history included the insertion of a copper IUD 20 years ago. The IUD has not been changed until its removal 1 month prior to the admission. No other relevant medical or surgical history was found besides smoking. Laboratory findings showed an increased C-reactive protein level (203 mg/l), leukocytosis (13,000/mm^3^) and microcytic anemia (hemoglobin level: 9.2 g/dl). The tumor markers were normal except the CA-125 which reached 209 mIU/l. Pelvic and abdominal computed tomography (CT) and MRI were performed and revealed bilateral ovarian multiloculated cystic lesions with multiple anterior pelvic implants in the utero-vesical space evoking peritoneal carcinomatosis. The largest implant above the bladder dome measured 2 cm, while the ovarian lesions reached 4 cm in diameter (Figure 1). Pelvic lymphadenopathies were also seen on the CT scan.

**Figure 2.**
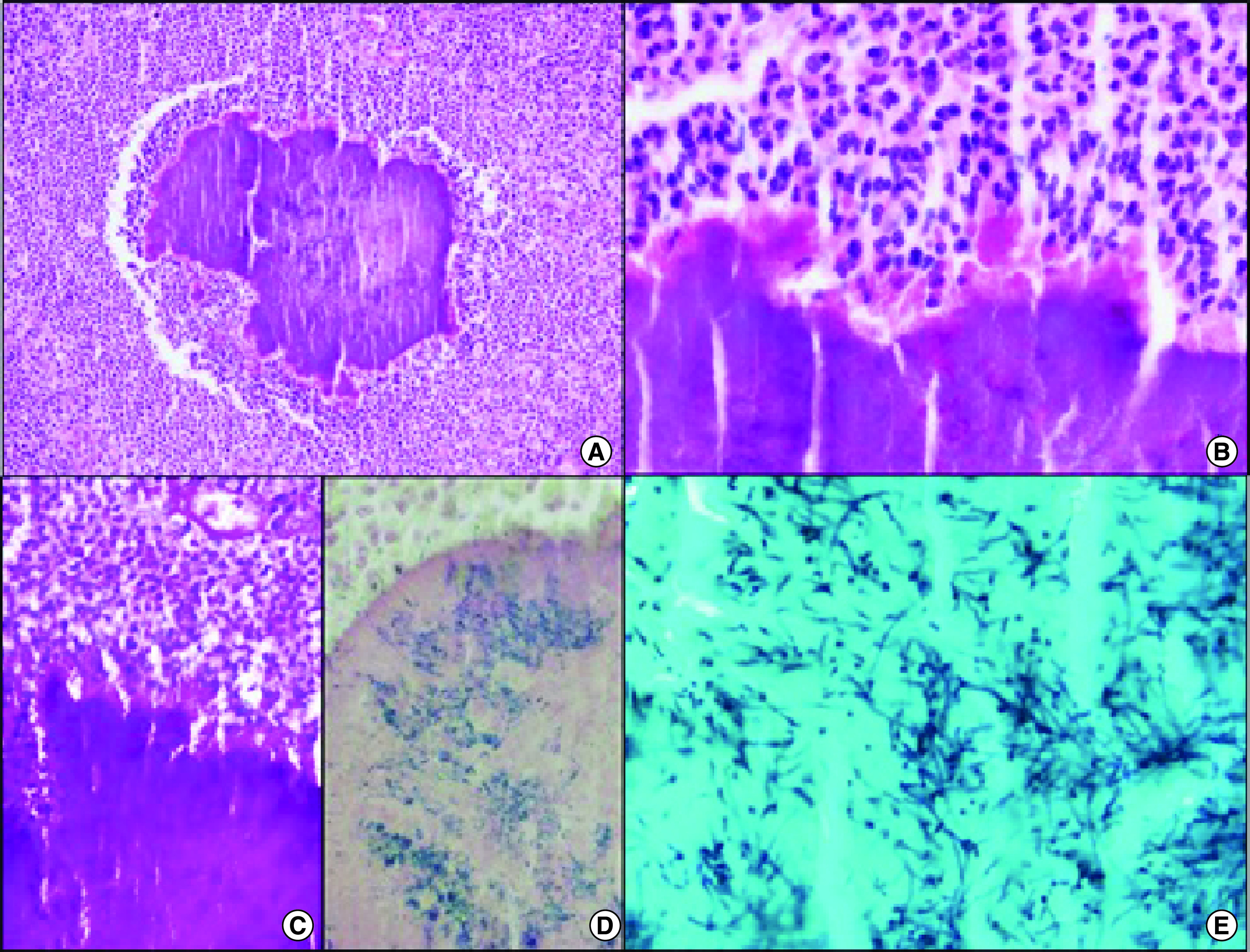
Histological findings of the surgical specimen. **(A & B)** The clumps of basophilic filamentous bacteria show a rosette-like configuration surrounded by a suppurative mixed inflammatory infiltrate (hematoxylin and eosin [H&E] ×100 and H&E ×400, respectively). **(C & D)** The bacterial clumps are Gram-positive and PAS- [periodic acid-Schiff] positive (PAS ×200 and Gram ×400, respectively). **(E)** A Gomori methenamine silver (GMS) stain highlights the filamentous shape of the bacteria (GMS ×1000).

The case was discussed in a multidisciplinary meeting at our institution and an exploratory laparoscopy with biopsies was recommended. The procedure showed severe adhesions in the lower abdomen with frozen pelvis and ascites. Due to the fear of spreading eventual malignant cells, the pelvic magma was not dissected or ruptured laparoscopically. In order to rule out malignancy, peritoneal biopsies were taken and ascites fluid was collected for cytological examination. Histological examination demonstrated that the peritoneal nodules were fibrosing and inflammatory. No malignant features were found. The patient was; therefore, discharged empirically on ciprofloxacin. Thus, the patient described a persistent pelvic pain and pressure resistant to analgesics 6 weeks later. We therefore, decided to undergo a surgical treatment due to the patient’s discomfort and the absence of bacterial growth on samples collected during the laparoscopy. An exploratory laparotomy was performed: an indurated nodule was seen in the vesico-uterine space and was invading the posterior bladder wall. Posteriorly, the adnexal magma was adherent to the ileocecal valve and infiltrated the sigmoid. Total abdominal hysterectomy with bilateral salpingo-oophorectomy was performed, associated with partial cystectomy, cecal resection, sigmoidal wedge resection and partial omentectomy. Visceral resection was limited due to the atypical inflammatory aspect that was seen macroscopically; though, it was performed in order to secure clear margins away from any potential malignant tissue. The bladder and sigmoidal defects were closed using continuous resorbable suturing. On macroscopic examination, thick yellowish purulent material was filling both ovaries. The histopathological examination revealed no evidence of malignancy; the ovaries showed foci of extensive suppurative granulomatous inflammation with no caseous necrosis, focally containing clumps of basophilic filamentous bacteria that were surrounded by acute inflammation and were positive for Gram, periodic acid-Schiff (PAS) and grocott methenamine silver (GMS) stains, consistent with actinomycosis (Figure 2). The same inflammatory foci were seen in the bladder wall, containing the clumps of filamentous bacteria. The endometrium and the fallopian tubes showed on the other hand signs of chronic endometritis/salpingitis with lymphocytic infiltrate.

The postoperative period was uncomplicated: a urinary catheter was left in place for 2 weeks and bowel movements returned on day 5 with progressive diet initiated thereafter. The patient received intravenous ampicillin during her hospital stay and was discharged on ampicillin 1 g daily for 6 months. Retrograde cystourethrogram returned normal 2 weeks after the surgery. The patient was followed-up afterward for 2 years with no recurrences detected so far.

## Discussion

Because of their low potential for virulence,* Actinomyces* require an alteration of the normal mucosa through trauma, surgery, infection or foreign body to cause the formation of abscesses and fistulae [2]. A systematic review of all the reported cases of pelvic actinomycosis between 1980 and 2014 demonstrated that the major initiating factor of pelvic infection is the use of IUDs [2]. An IUD induces fistulations of the endometrial mucosa, leading to the entry of *Actinomyces* bacteria into the submucosal space where the chronic infection takes place and disseminates, culminating in the formation of the pseudo-tumor abscess [3,4]. The diagnosis should be considered in patients that wore IUDs for prolonged periods, especially more than 5 years and should not be confounded with colonization of IUDs by *Actinomyces*. In our case, since the infection affected mainly the ovaries, we suppose that the long-disposed IUD has led to the growth of *Actinomyces* micro-organisms through its wires, culminating in an ascending infection that reached the adnexa. The infection disseminated throughout the tubes and caused a destruction of the ovarian parenchyma. From this point, bladder, ileocaecum and the rectosigmoid were also affected. To the best of our knowledge, few of the cases reported in the literature have involved both ovaries as well as the bladder and rectal wall, requiring partial cystectomy for diagnostic purposes after cultures returned negative. Reported cases of bladder actinomycosis have underwent transurethral biopsies and drainage showing only chronic inflammation, since the lesion is not intraluminal but infiltrates the bladder from the serosal layer inwards [5–9]. In our case, the patient did not present with lower urinary tract symptoms, which was the reason why cystoscopy and transparietal biopsies were not performed.

*Actinomyces* are slow-growing and tissue-invading bacteria; therefore, pelvic infection can imitate a carcinogenic process: suppurative abscesses and granulomas take months to form, with the most common symptoms being a progressively enlarging pelvic mass, weight loss and pelvic pain. Pelvic actinomycosis can also lead to an elevation of CA-125 levels [2,3], as seen in our case. The infection usually mimics malignancy since the disease is infiltrative and can invade separate tissue layers and organs; the diagnostic images including CT and MRI cannot distinguish between actinomycosis and malignant lesions: they can help along with laparoscopy in determining the degree of dissemination [2]. In most cases, the diagnosis of actinomycosis is established posteriorly on the surgical specimen obtained after the performance of a laparotomy or a laparoscopy to evaluate the suspicious pelvic mass [2].

Treatment of actinomycosis can be completed using antibiotic therapies as simple as penicillins or lincosamides, for 6–12 months [10]. Short treatment courses are thought to be ineffective and increase the risk of relapses [2]. Draining the abscesses and resecting the injured tissue is performed by some surgeons when clinical and radiological findings do not resolve after 4–6 weeks of antibiotic therapy. However, surgical management should be reserved for urgent cases with acute abdomen, intestinal or ureteral obstruction, intractable sinuses and in cases with an unreached diagnosis [11]. It has been shown that genitourinary actinomycosis usually requires high doses of prolonged intravenous antibiotics when compared with the other forms of the disease [12]. Although pelvic actinomycosis usually presents in advanced stages due to the nature of the disease not allowing early diagnosis, the disease can be completely cured by medical therapy alone for a prolonged period of time. Even large infiltrates can be turned into insignificant fibrous scars after long-term antibiotic therapy. Since it removes the burden of the infected tissue at once, surgery, as seen in our case, can accelerate disease resolution when compared with antibiotic therapy, but should not be recommended; also, when extensive surgical resection is performed, the duration of antibiotic therapy should be reduced [12].

In this paper, we highlighted the confusion that can occur between pelvic actinomycosis and malignant lesions. An erroneous clinical diagnosis has been reported frequently in the literature [2,10]. This highlights the importance of using appropriate diagnostic tools when facing tumor-like pelvic lesions in patients with forgotten IUDs, in order to identify the causative agent and undergo appropriate medical treatment, preventing diagnostic error that could lead to unnecessary invasive treatment. Bacteriological cultures have been found to be of little help since they return negative in a high proportion of cases [4,5]. Recently, new diagnostic tools are being used: these methods include biochemical testing and germ identification through RNA sequencing [4].

In conclusion, this paper demonstrates that ovarian actinomycosis can be accompanied by supra-vesical abscesses with infiltration of the bladder dome. In such advanced disease, there is a benefit in applying highly sensitive diagnostic tools in order to reach the correct diagnosis and prevent the patient from undergoing extensive visceral resection which can harvest adverse outcomes without affecting the risk of relapse on long term follow up.

## Future perspective

We believe that research will focus in the upcoming years on new diagnostic tools that allow identification of the *Actinomyces* spp. with high sensitivity, allowing the detection of the germ in a considerable proportion of cases. More evidence to support the use of biochemical testing and RNA sequencing in this field is required. Furthermore, long-term safety data should be developed concerning the use of IUDs for more than 5 years. Risk factors for the occurrence of pelvic actinomycosis in women with IUDs should be studied, and early replacement of the device should be recommended in such patients.

**Executive summary**

- Pelvic actinomycosis is a malignancy-mimicking disease that can arise in genital tracts where intrauterine devices are kept in place for more than 5 years.
- The disease can be cured in general with high dose intravenous antibiotic therapy for 1 month followed by prolonged treatment with oral antibiotics for 6–12 months.
- Surgery is reserved for cases with acute complications such as fistulas, intestinal occlusions and peritonitis.
- Guided biopsies should be performed in cases suspected on history, clinical exam and biology results in order to prevent unnecessary surgical interventions.
- Special techniques can be used if regular cultures return negative. Using specific culture medias allowing the growth of *Actinomyces* spp. is paramount.
- Awareness among gynecologists and women is important in order to seek replacement of their intrauterine device every 5 years.
